# Everyday Racial Discrimination and Hypertension among Midlife African American Women: Disentangling the Role of Active Coping Dispositions versus Active Coping Behaviors

**DOI:** 10.3390/ijerph16234759

**Published:** 2019-11-27

**Authors:** Eli K. Michaels, Alexis N. Reeves, Marilyn D. Thomas, Melisa M. Price, Rebecca E. Hasson, David H. Chae, Amani M. Allen

**Affiliations:** 1 Division of Epidemiology, University of California Berkeley School of Public Health, 2121 Berkeley Way #5302, Berkeley, CA 94720-7360, USA; alexisnicolereeves@berkeley.edu (A.N.R.); marilyn.thomas@berkeley.edu (M.D.T.); 2 Division of Community Health Sciences, University of California Berkeley School of Public Health, 2121 Berkeley Way #5302, Berkeley, CA 94720-7360, USA; melisa.price@ucsf.edu; 3 Schools of Kinesiology and Public Health, University of Michigan, 2110 Observatory Lodge/1402 Washington Heights, Ann Arbor, MI 48109-2029, USA; hassonr@umich.edu; 4 Department of Human Development and Family Studies, College of Human Sciences, Auburn University, 210 Spidle Hall, Auburn, GA 36849, USA; david.chae@auburn.edu; 5 Divisions of Community Health Sciences and Epidemiology, University of California Berkeley School of Public Health, 2121 Berkeley Way #5302, Berkeley, CA 94720-7360, USA; amaniallen@berkeley.edu

**Keywords:** racial discrimination, hypertension, stress, coping, John Henryism, African American, health disparities

## Abstract

Racial discrimination, a psychosocial stressor, may contribute to disproportionate rates of hypertension among African American women. Coping moderates the effects of psychosocial stress on health. Coping dispositions describe stable personality characteristics, whereas contextual frameworks emphasize flexible coping behaviors in response to specific stressful encounters. Using data from the African American Women’s Heart and Health Study—a non-probability cross-section of 208 midlife African American women in Northern California—we estimated the association between everyday racial discrimination (Everyday Discrimination Scale, EDS) and prevalence of hypertension (HTN), and evaluated moderation by coping disposition (John Henryism Active Coping scale, JH) versus context-specific active coping behavior (Active Coping with Racism scale, ACR). There were no main associations between EDS, JH, or ACR on HTN prevalence. There was evidence of statistical interaction between EDS and ACR (p-int = 0.05), but not JH (p-int = 0.90). Among those with high levels of ACR, reporting monthly (prevalence ratio (PR) = 2.35, 95% confidence interval (CI) = 1.13, 4.87), weekly (PR = 2.15, 95% CI = 1.01, 4.61), or daily (PR = 2.36, 95% CI = 1.14, 4.88) EDS was associated with higher HTN prevalence, versus reporting racial discrimination yearly or less. In contrast, among those with low levels of ACR, reporting more chronic racial discrimination was associated with lower hypertension prevalence, although results were less precise. Findings suggest that ongoing active coping with chronic racial discrimination may contribute to hypertension risk among African American women.

## 1. Introduction

Hypertension is a primary risk factor for cardiovascular disease [1,2], the leading cause of death among African American women, and the largest driver of the Black-White life expectancy gap in the United States [3,4]. Over half (53.2%) of non-Hispanic African American women suffer from hypertension, compared to 42.8% among women of all racial groups and 38.8% among non-Hispanic White women [5]. Moreover, African American women experience earlier onset and more rapid progression of hypertension and other chronic health conditions compared to other racial and gender groups, a phenomenon referred to as accelerated aging, or *weathering* [6,7]. The determinants of racial inequities in hypertension incidence, prevalence, and progression are not fully understood, as they often persist after accounting for myriad socioeconomic, behavioral, and biomedical factors [8,9,10].

The *weathering hypothesis* posits that repeated and ongoing adaptation to structural marginalization and associated psychosocial stress contributes to accelerated physiologic aging among African American women and other stigmatized groups [6,11]. Stress is defined as the process by which social and environmental demands exceed one’s perceived capacity to effectively cope with the demands [12]. This experience elicits both behavioral and physiological responses, which can lead to increased and prolonged activation of the sympathetic nervous system [13,14,15,16], a primary pathway to blood pressure dysregulation [17].

Racial discrimination—or the experience of unfair treatment on the basis of race, ethnicity, or skin color—is a pervasive and salient psychosocial stressor commonly reported by African American women [18,19,20,21,22] that has been implicated in the higher rates of hypertension among this group, as well as among African American men and Black populations more broadly [10,23,24,25,26,27,28,29]. However, despite what we know about the stressfulness of racial discrimination, and the role of stress in the pathogenesis of hypertension, findings of an association between self-reported discrimination and myriad cardiovascular indicators are mixed [23,25,26,28,29,30]. Some studies have demonstrated evidence of associations between general or racial discrimination and various cardiovascular outcomes among African Americans, including high blood pressure or hypertension [31,32,33,34,35,36], increased cardiovascular reactivity [37,38,39,40], and lower nocturnal blood pressure dipping [41], whereas others have shown null effects [24,42,43,44,45]. Moreover, rather than consistently showing a linear or dose-response relationship, previous literature has illustrated distinctive patterns of association between frequency of racial discrimination and cardiovascular and other stress-related outcomes among African Americans (e.g., J-shaped, U-shaped, inverse U-shaped, M-shaped) [34,35,36,43,46,47,48,49].

One possible contributor to mixed and complex findings in the racial discrimination and cardiovascular health literature is the omission of coping, a central component of the stress-response process [19,25,27,50,51]. Coping has been defined as “changing cognitive and behavioral efforts to manage specific external and internal demands that are appraised as taxing or exceeding the resources of the person” [52] (p. 141). According to stress theory, the physiologic consequences of stress exposure will depend on one’s cognitive and behavioral coping response [14,50,52]. Moreover, it has been suggested that an individuals’ willingness to self-report sensitive information—such as exposure to racial discrimination—may be influenced by coping style, potentially contributing to poorer health outcomes among those who report no discrimination experiences [30,34,35,46,47,53]. Accordingly, scholars have recommended integrating coping to more fully elucidate relationship between racial discrimination and health outcomes among African Americans [19,27,50,51]. However, the current literature lacks consensus about the appropriate conceptualization and measurement of coping in this area of work [50].

There are several dominant paradigms in the coping literature. Some conceptualize coping as a relatively stable personality trait, irrespective of the type of stress encountered (e.g., how do you normally manage stress?) [54,55,56,57,58,59,60,61,62]; others view coping as a flexible behavior that manifests according to the specific nature of the stressor (e.g., what did you do the last time you and your spouse got into an argument?) [19,50,52,63,64,65]. Importantly, the two constructs are not strongly correlated, in that one’s general coping disposition may not reliably predict their coping responses across varied stressful contexts [63]. Arguing that both constructs are needed to fully understand the coping process, Moos and Holihan developed the integrated *dispositional and contextual coping perspectives* framework, in which they summarize: “the concept of coping encompasses relatively stable coping styles or dispositions that characterize individuals’ habitual interactions with their environments *as well as* the cognitive and behavioral coping responses or skills individuals employ to manage specific stressful encounters” [65] (pp. 1387–88). Similarly, with respect to racism-related stress, Clark and colleagues distinguish “general coping responses” from “racism-specific coping responses” [19] (pp. 809–810). For example, in previous qualitative work, African American women described using unique coping strategies in relation to racial discrimination compared to other forms of psychosocial stress [18]. Hence, racism-related stress may elicit unique coping behaviors (i.e., contextual) that differ from general coping tendencies (i.e., dispositional), and differentiating between the two forms of coping may improve understanding of how racism as a psychosocial stressor impacts health for African Americans [18,19,65].

John Henryism is one example of a *coping disposition* that is often studied in relation to cardiovascular health outcomes among African Americans [54,55,56,57,58,59,60,61]. Based on the legend of John Henry—the “steel-driving man” with great physical prowess who succeeded at racing a steel-driving machine in driving nails into a railroad track, but immediately died of physical exertion—John Henryism can be defined as “a strong behavioral predisposition to cope actively with psychosocial environmental stressors” [58] (p. 163). Importantly, the John Henryism disposition is structurally- and historically-rooted [66], reflecting “the larger protracted struggle of African American men and women…to free themselves from pervasive and deeply entrenched systems of social and economic oppression” [58] (p. 167). The *John Henryism hypothesis* posits that for African Americans experiencing social and economic adversity, the John Henryism coping disposition will increase risk of hypertension and other adverse cardiovascular outcomes [58,59]. In support of this hypothesis, previous studies have found evidence of interaction between racial discrimination and John Henryism whereby chronic exposure to racial discrimination, coupled with higher levels of John Henryism, are associated with higher risk of high blood pressure [67,68] and other adverse health outcomes [57,69] among African Americans.

Taking a more *contextual coping* approach, several studies have focused explicitly on racism-specific active coping behavior as a moderator of the association between racial discrimination and hypertension among African American women [34,35]. Using data from the Coronary Artery Risk Development in Young Adults (CARDIA) study, Krieger and colleagues found that most (70–75%) African American women coped actively with racial discrimination by “trying to do something and talking to others about it” [34,35]. Authors found that active coping attenuated the effects of racial discrimination on blood pressure among African American women: Those who reported no racial discrimination and coped passively by “accepting it as a fact of life and keeping it to themselves” had higher blood pressure compared to those who reported any racial discrimination and coped actively [34,35].

While both John Henryism, an active coping disposition, and racism-specific active coping behaviors have been shown to moderate the association between racial discrimination and cardiovascular health outcomes among African American women [34,35,67,68], the two constructs should not be conflated [65,69]. As described above, habitual coping tendencies do not reliably predict context-specific coping behaviors across various kinds of stressful encounters [63], and racism-related stressors may elicit distinct coping behaviors that may be incongruent with one’s general coping tendencies [18,19]. Therefore, the common implicit assumption that those with a higher John Henryism disposition necessarily are coping actively with racial discrimination and related stress may be misguided. Moreover, previous evidence suggests that the John Henryism active coping disposition may exacerbate the health consequences of racial discrimination [67,68], whereas racism-specific active coping behavior may buffer its effects [34,35]. Thus, the John Henryism active coping disposition and racism-specific active coping behaviors are distinct constructs that may function differentially in the stress-response process.

To our knowledge, only one study distinguished John Henryism as a coping *disposition* from how individuals manage racism-specific stressors [69]. Authors found that John Henryism attenuated the positive association between racial discrimination and depressive symptoms among African American men; however, this interaction was only evident among men who tended to cope actively with racial discrimination-specific stress [69]. Findings underscore the utility of examining general coping dispositions (e.g., John Henryism) *and* context-specific coping behaviors (e.g., racism-specific active coping) as unique constructs that may independently and interactively modify the health consequences of racial discrimination. No studies, to our knowledge, have attempted to disentangle these constructs in relation to racial discrimination and hypertension among African American women, a group facing both racial discrimination and adverse cardiovascular health at disproportionate rates [5,6,7,18,22]. A better understanding of whether active coping dispositions and context-dependent active coping behaviors correlate with each other and how they may function similarly or differentially in the stress-response process can be used to refine etiologic hypotheses and inform targeted interventions for African American women navigating experiences of racial discrimination.

Therefore, the present study aimed to assess the relationship between John Henryism coping disposition and racism-specific active coping behavior and whether either or both moderated the association between racial discrimination and hypertension among a community sample of midlife African American women. Findings showed a weak correlation between John Henryism and racism-specific active coping behavior, confirming the notion that these are distinct psychosocial constructs that should not be conflated. Active coping with racism, but not John Henryism, moderated the association between racial discrimination and hypertension. Specifically, among African American women who tended to cope actively with racism, reporting more chronic experiences of racial discrimination was associated with a higher prevalence of hypertension compared to reporting less chronic experiences.

## 2. Materials and Methods

### 2.1. Study and Recruitment

Data are from the African American Women’s Heart and Health Study, which was designed to explore relationships between environmental exposures, psychosocial stressors, and various mental and physical health outcomes among a cross-sectional, community sample of midlife African American women aged 30–50 residing in four San Francisco, Bay Area counties (n = 208).

The study protocol is described in more detail elsewhere [47]. Briefly, purposive sampling and targeted recruitment were used to ensure a socioeconomically heterogeneous sample of African American women. Eligibility criteria included: (1) self-identified African American, (2) female since birth, (3) age 30–50, (4) US-born, (5) parent(s)/primary caregiver(s) US-born African American, (6) could read/write English. Exclusion criteria included: (1) pregnant or lactating and/or (2) self-reported a physician-diagnosed inflammatory or auto-immune disease. Data collection included an interviewer-administered questionnaire and computer-assisted self-interview for more sensitive topics, as well as a physical examination including anthropometric measurements and blood pressure assessment. All subjects gave their informed consent for inclusion before they participated in the study. The study was conducted in accordance with the Declaration of Helsinki, and the protocol was approved by the Committee for the Protection of Human Subjects at the University of California, Berkeley (2010-09-2240).

### 2.2. Study Measures

#### 2.2.1. Hypertension

Resting diastolic and systolic blood pressure were assessed as the average of three consecutive measurements from a seated position using an automated blood pressure monitor [70]. Following the most recent American College of Cardiology and American Heart Association guidelines, hypertension (HTN) was defined as: (a) systolic blood pressure ≥ 130 mmHg or (b) diastolic blood pressure ≥80 mmHg or (c) self-reported current cardiovascular medication use [5,71].

#### 2.2.2. Everyday Racial Discrimination

Racial discrimination was assessed using a modified version of the Everyday Discrimination Scale (EDS) [72]. Respondents were asked to report the frequency of racial discrimination across ten social situations: “In your day-to-day life, how often have the following things happened to you because of your race, ethnicity, or skin color?” Examples include: “people act as if they’re better than you are,” “people act as if they think you are not smart,” and “you are followed around in stores” (Supplementary Table S1). Six response categories captured the frequency of experiences ranging from “never” to “almost everyday” (α = 0.95).

Because racial discrimination is believed to impact hypertension through repeated biological adaptation to chronic psychosocial stress [10,27], precisely and accurately measuring the chronicity of these experiences was important for valid exposure assessment in this study [36]. A recent study compared three different approaches to coding the EDS, finding that conventional coding schemes may fail to fully quantify the chronicity of racial discrimination, leading to exposure misclassification and an underestimation of associations with hypertension among African American women [36]. Therefore, following prior work, we weighted each EDS survey item to more accurately reflect the chronicity of experiences on a yearly scale: never = 0, less than once a year = 0.5 times per year (x/year), a few times a year = 3 x/year, a few times a month = 3 x 12 months = 36 x/year, at least once a week = 2 x 52 weeks = 104 x/year, and almost every day = 5 x 52 weeks = 260 x/year (Table 1) [36]. Recoded items were summed to generate a score ranging from 0–2600, representing the total annual number of EDS experiences across ten situations.

As detailed in Table 1, we then categorized the EDS summary score into five levels to represent experiencing racial discrimination yearly or less, monthly, weekly, daily, or hourly. Yearly or less EDS (lowest reported frequency of racial discrimination) was used as the referent category for all regression analyses. The rationale for creating these categories was twofold: first, to preserve the qualitatively meaningful, time-bound structure of the original scale (e.g., yearly, monthly, daily); second, to capture potentially differential associations with hypertension among those experiencing varying levels of racial discrimination chronicity (e.g., on a yearly versus daily basis) without imposing a specific functional form (e.g., linear, quadratic) on the relationship between racial discrimination and hypertension prevalence. Our approach is consistent with previous studies that used a categorical classification of racial discrimination and showed varying patterns of association with health outcomes among African Americans [36,43,46,47,48].

#### 2.2.3. John Henryism

John Henryism (JH) was assessed via the John Henryism Active Coping (JHAC12) scale, a validated 12-item scale capturing disposition toward active, high-effort coping with social-environmental stressors [56,59]. JHAC12 captures general dispositional tendencies, rather than concrete behaviors in context-specific situations (e.g., work or relationship stress, racial discrimination). Sample items include: “when things don’t go the way I want them to, that just makes me work even harder,” “in the past, even when things got really tough, I never lost sight of my goals,” shown in Supplementary Table S2. Responses range from 1 “completely false” to 5 “completely true” (α = 0.85). Items were summed to create a score with higher values reflecting a stronger JH disposition.

#### 2.2.4. Active Coping with Racism

Active Coping with Racism (ACR) is a composite variable developed to capture the degree to which respondents cope in an active, effortful way when confronted with racism-specific stressors. Respondents were asked: “Please indicate how often you do each of the following when you are treated unfairly because of your race, ethnicity or skin color.” Three items (“speak up or try do something about it,” “work harder to try to change the situation,” and “work harder to try to prove them wrong”) were selected for a composite variable because they represented racism-specific coping behaviors most congruent with a John Henryism disposition (Supplementary Table S3). Response options range from 1 “never” to 4 “most of the time.” A polychoric correlation analysis [59] of the three active coping behaviors revealed moderate-to-strong covariance between items (range: 0.48–0.67) and a unidimensional data structure, with 70% of the variance explained by the first component (eigenvalue = 2.10). The alpha test demonstrated acceptable internal consistency (α = 0.74). The three ACR items were summed to generate a summary ACR score ranging from 3 (i.e., “never” on all three items) to 12 (i.e., “most of the time” on all three items).

#### 2.2.5. Covariates

Covariate selection was based on theory and existing literature on potential confounders of the association between racial discrimination and hypertension [35,54,55,69]. All covariates were self-reported, except for body mass index (BMI), which was calculated as weight (kg)/height (m)^2^ based on anthropometric readings. Age was modeled continuously and all other covariates were dichotomized so that a higher value corresponds with the higher risk category for hypertension: not married/partnered, ≤100% federal poverty level, ≤high school diploma, unemployed, BMI ≥ 25 or <18.5, ≥3 alcoholic drinks per day, exercise < 5 times/week, and current smoker (Table 1). Bivariate associations between all study variables are displayed in Supplemental Table S4.

### 2.3. Analysis

We performed multiple imputations to account for missing data. A series of bivariate analyses comparing the distribution of covariates between participants with and without missing data suggested data were likely missing at random, a required assumption for multiple imputations [73,74]. Active coping with racism and blood pressure had the highest frequency of missing information (6.76% and 4.83%, respectively). We log-transformed systolic and diastolic blood pressure to meet the normality assumptions of multiple imputations [74]. One study participant was missing prediction data and was therefore excluded from the analytic sample prior to imputation (n = 207). Relative variance increase was <10% for all models and relative efficiency ranged from 98% to 100% [74].

Pearson’s correlation coefficients were generated to evaluate the association between John Henryism coping disposition and Active Coping with Racism. Next, we used modified Poisson regression models with robust standard errors to estimate hypertension prevalence ratios (PRs) and 95% confidence intervals (CIs) as a function of EDS, JH, ACR, and interactions between EDS and JH and between EDS and ACR, adjusting for demographic, socioeconomic, biologic, and behavioral factors [75]. When the prevalence of a study outcome is not rare (>10%), as is the case with hypertension in our sample (54%) the odds ratio produced by a logistic regression will over-estimate the prevalence ratio and can lead to misleading study conclusions [75]. Therefore, we instead estimated the prevalence ratio, a more appropriate and conservative measure of association in this context.

We first modeled main associations between EDS and HTN (Model 1), JH and HTN (Model 2), and ACR and HTN (Model 4). We used a nested modeling approach to adjust for groups of potential confounders. Minimally-adjusted models included basic demographics (age and marital/partnership status). Partially-adjusted models added socioeconomic factors (poverty status, education, and employment) to the minimally-adjusted models. Fully-adjusted models added biologic and behavioral risk factors for hypertension (BMI, smoking, drinking, and exercise) to the partially-adjusted models. We report the fully-adjusted estimates here, however results were similar for all three adjustment sets. Point estimates for all model covariates are provided in Supplemental Tables S5 and S6; however, these estimates are not adjusted for confounders of the covariate-hypertension associations and therefore should be interpreted with caution [76].

Next, multiplicative interactions between EDS and the two coping scales were evaluated using EDS*JH (Model 3) and EDS*ACR (Model 5) interaction terms, and following the aforementioned stepwise modeling approach. JH and ACR were mean-standardized to avoid extrapolation problems and facilitate interpretation. We included a post-estimation overall joint test of significance for the interaction terms. It has been suggested that type II error is of greater concern than type I error when evaluating statistical interaction due to the possibility of “wrong model bias” resulting from the mistaken elimination of interaction effects [77]. Accordingly, we used a 10% alpha (*p* ≤ 0.10) threshold to evaluate statistical interaction. Finally, we used Stata’s lincom command to assess the association between EDS and hypertension at the minimum, median, and maximum levels of active coping with racism (ACR = 3, ACR = 9, and ACR = 12, respectively), given evidence of statistical interaction. This approach is akin to simple slopes analysis [78,79] with a binary outcome [80]. All analyses were performed using Stata SE v13 [81].

## 3. Results

### 3.1. Sample

The study sample characteristics are displayed in Table 2. The mean age was approximately 42 years. The majority of participants were not married or partnered, had more than a high school education, were not living in poverty, and were employed. Most participants were nonsmokers or former smokers, irregular exercisers, light drinkers, and had a BMI that was <18.5 or ≥25, outside of the recommended range for cardiovascular health [5]. The mean systolic blood pressure was 122 mmHg and the mean diastolic blood pressure was 80 mmHg. Less than half of participants reported taking cardiovascular medication. Using the most recent guidelines, over half of the study sample (54%) met the criterion for hypertension, comparable to the national prevalence among African American women (53%) [5].

### 3.2. Everyday Discrimination (EDS)

The distribution of responses to each EDS item may be found in Supplementary Table S1. Responses were summed across all ten items to create an annual EDS score ranging from 0 to 2600 with mean (SD) = 474 (695) total annual EDS experiences, corresponding to more than one discrimination experience per day (because 474 > 375). As shown in Table 2, respondents were distributed fairly evenly across the five EDS levels, with the largest proportion reporting experiencing some form of everyday racial discrimination either yearly or less (23%) or weekly (24%).

### 3.3. Coping

The distribution of responses to John Henryism and Active Coping with Racism questionnaire items are presented in Supplementary Table S2 and Supplementary Table S3, respectively. The JHAC12 summary score ranged from 16 to 60 with mean (SD) = 50 (7). As shown in Table 2, the ACR summary score ranged from 3 to 12, with mean (SD) = 9 (2). The correlation between John Henryism and active coping with racism was weak (r = 0.26) [82].

### 3.4. Regression Results

Table 3 displays the main associations between EDS and hypertension prevalence (Model 1), JH and hypertension (Model 2), and the interaction between EDS and JH on hypertension prevalence (Model 3). We did not find evidence of main associations between EDS or JH with hypertension prevalence, nor for their interaction (p-int = 0.90).

Table 4 displays the main association between ACR and hypertension prevalence (Model 4) and the interaction between EDS and ACR on hypertension prevalence (Model 5). Model 4 shows a null main association between ACR and hypertension prevalence. Model 5 shows a significant EDS*ACR interaction (p-int = 0.05).

### 3.5. Simple Slope Analysis

**Figure 1:**
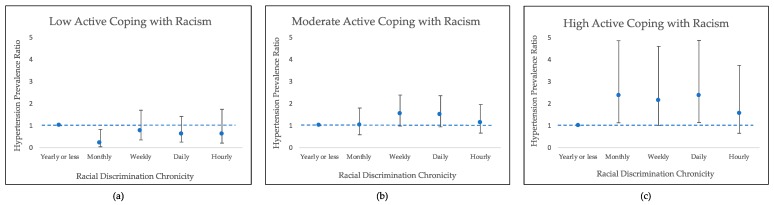
Prevalence ratios (PRs) and 95% confidence intervals (CIs) for associations between everyday racial discrimination (Everyday Discrimination Scale) and hypertension at (**a**) minimum (ACR = 3); (**b**) median (ACR = 9); and (**c**) maximum (ACR = 12) values of Active Coping with Racism, adjusting for: age, marital/partnership status, income, education, employment, BMI, smoking, drinking, and exercise, African American Women’s Heart and Health Study (n = 207).

Table 5 and Figure 1 show hypertension prevalence ratios and 95% CIs comparing each level of EDS to the referent category (yearly or less), when Active Coping with Racism is fixed at its minimum (ACR = 3), median (ACR = 9), and maximum (ACR = 12) values.

Among those with moderate tendencies toward active coping with racism (i.e., when ACR = 9, the median), the association between racial discrimination and hypertension was null. However, distinct patterns of association emerged among those with the lowest and highest ACR scores. Among those who did not tend to cope actively with racism (i.e., when ACR = 3, the minimum), reporting monthly, weekly, daily, or hourly EDS was associated with a lower prevalence of hypertension compared to reporting yearly or less frequent EDS. The prevalence ratio comparing monthly vs. yearly EDS was most precise (PR [95% CI] = 0.20 [0.05, 0.83]), whereas the other estimates contained the null. In contrast, among highly active copers (i.e., when ACR = 12, the maximum), reporting monthly, weekly, or daily EDS was significantly associated with twofold higher prevalence of HTN compared to reporting yearly EDS or less.

## 4. Discussion

This is the first study, to our knowledge, to compare the John Henryism active coping disposition and racism-specific active coping behavior as moderators of the association between racial discrimination and hypertension among African American women. Four key findings emerged.

First, the main association between everyday racial discrimination and hypertension was null, consistent with previous work [24,42,43,44,45]. It has been suggested that omitting coping as a key moderator may obscure associations between racial discrimination and adverse health outcomes; our finding supports this notion [27,50,69]. At the same time, our finding conflicts with work showing significant main associations between racial discrimination and high blood pressure or hypertension among African Americans [31,32,33,34,35,36]. Mixed findings may be related to sample differences or inconsistencies in measurement of the exposure and outcome, among other methodological distinctions [27,28,36,46,83].

Second, John Henryism active coping disposition and racism-specific active coping behavior were weakly correlated and showed adifferential modification of the association between racial discrimination and hypertension. Thus, while John Henryism may be associated with a general disposition toward active coping [56], it may not directly translate into coping actively with racism-specific stressors [19,63,65,69]. This finding is consistent with Moos and Holihan’s *dispositional versus contextual coping perspectives* framework, supporting the need to delineate these constructs to better understand how African American women navigate racial discrimination and related stress [65]. In addition, we found evidence of interaction of racial discrimination with racism-specific active coping behavior, but not with John Henryism. This finding suggests that contextual coping behavior may play a more salient mechanistic role than general coping disposition in the relationship between racial discrimination and hypertension among African American women. This interpretation is consistent with Kemeny’s *integrated specificity* model, which posits that the biological stress response reflects a combination of an individual’s stressor-specific cognitive appraisal and coping behavior [51]. Hence, different kinds of stressors experienced among African American women may elicit a range of context-specific coping strategies, which may produce distinct biological states with varied implications for health [18,51,84].

Third, among women with a greater tendency toward active coping with racism (i.e., high ACR), reports of experiencing racial discrimination monthly or more was associated with a higher prevalence of hypertension compared to reports of experiencing racial discrimination yearly or less. One plausible interpretation is that racial discrimination is such a powerful source of psychosocial stress among African American women, its effects can only be attenuated when it is experienced infrequently among those who deploy highly active coping techniques. More chronic racial discrimination, even when confronted with active coping strategies, may be associated with higher hypertension prevalence. Put differently, racism-specific active coping behavior may be adaptive for managing acute instances of racial discrimination, but may be ineffective or even health-damaging when discrimination is more chronic and potentially appraised as uncontrollable [19,50,64,85,86,87]. This finding supports the *weathering hypothesis* that persistent high-effort coping with chronic psychosocial stress may cause wear and tear on the body’s regulatory systems, increasing chronic disease risk [16,19,59,88].

Our findings among those with higher racism-specific active coping tendencies (i.e., high ACR) were also consistent with the *John Henryism hypothesis* [58,59], and with results from several studies documenting interactions between John Henryism and racial discrimination on blood pressure among African Americans [67,68]. One study of Black college students found that among those with higher levels of John Henryism, more frequent reports of racial discrimination were associated with higher resting diastolic blood pressure [68]; this aligns with our finding that higher levels of racism-specific active coping, coupled with more chronic experiences of racial discrimination, were associated with higher hypertension prevalence. Others found that John Henryism lowered blood pressure reactivity among African American women exposed to low levels of racism [67], consistent with our finding of lowest hypertension prevalence among women who tended to cope actively with racism and who reported experiencing racial discrimination yearly or less. Paradoxically, our results were unique to racism-specific active coping behavior whereas there were no notable findings for John Henryism coping disposition. One possible explanation is that the congruence between the John Henryism and racism-specific active coping behavior was stronger in these previous studies than in our sample of midlife African American women due to differences in age, geography, socioeconomic status, or other factors. Further research is needed to evaluate for whom, and under what conditions, a John Henryism disposition predicts active coping behavior across various kinds of stressful situations.

Fourth, among those who rarely coped actively with racism (i.e., low ACR), reporting racial discrimination yearly or less was associated with the highest prevalence of hypertension, whereas reporting more chronic exposure was associated with lower hypertension prevalence. Of note, confidence intervals among those with low levels of ACR were wide and should, therefore, be interpreted as hypothesis-generating. One hypothesis is that women with low active coping with racism tendencies are more likely to cope passively and engage in suppression, denial, or self-blame, which may lead to underreporting of racial discrimination and poorer health outcomes [18,35,53,80,89,90,91]. Indeed, previous work found higher blood pressure among African American women in the CARDIA study who reported never experiencing racial discrimination and who coped passively by “accepting it as a fact of life” and “keeping it to themselves” [34,35]. For these passive copers, reporting experiences of racial discrimination may be health-promoting because it necessitates acknowledging and externalizing racial discrimination, rather than internalizing and suppressing experiences of unfair treatment [80,90]. Such externalization has been linked with lower risk of hypertension and other health outcomes among African Americans [34,35,89,90]. Findings warrant further consideration with larger samples and explicit measurement of both active and passive coping.

This study had several notable strengths. The within-group study design and targeted recruitment strategy maximized heterogeneity of sociodemographic factors among a diverse sample of midlife African American women. Rather than treating this group as a monolith, our approach acknowledges and interrogates the diversity of African American women’s life experiences, dispositions, behaviors, and health states [18,92,93]. The collection of detailed socioeconomic, behavioral, and health information facilitated comprehensive confounder adjustment, and the use of well-validated and internally consistent scales for racial discrimination and John Henryism strengthened internal validity.

There were also limitations. Data are from a non-probability sample in Northern California. Results are not intended to be generalizable, but rather to explore potential interactions between psychosocial stressors and coping responses that can inform hypotheses regarding the mechanisms through which racial discrimination may harm health for African American women. Due to the exploratory nature of this study, our sample size was too small to conduct several subgroup analyses of interest. For example, evidence suggests that both John Henryism and active coping behavior interact with socioeconomic position to impact health [34,35,59,60,61]. Others showed differential interactions between racial discrimination and John Henryism among those who coped actively with racism versus those who did not [69]. We were underpowered to stratify the analysis on income or education, or to examine three-way interactions between racial discrimination, John Henryism, and racism-specific active coping behavior, potentially masking variation in results. Such stratified analyses are an important area for future work that can inform targeted interventions. Lastly, our final models adjusted for health behaviors and BMI, which could be mediators on the pathway from racial discrimination to hypertension [23]. Because the cross-sectional analysis precludes assessing mediation, we included these variables to provide conservative estimates. Future research using longitudinal data structures can more formally evaluate mediating relationships.

Future research may also consider extending the present work to include a wider range of coping behaviors and more diverse samples. The present analysis focused explicitly on racism-specific active coping behavior (e.g., “work harder to try to change the [racist] situation”) and compared it to John Henryism, a general disposition toward active coping (not necessarily in relation to racism). However, African American women may use a variety of behaviors—independently or simultaneously—to cope with racial discrimination, including but not limited to: active, passive, problem-focused, and emotion-focused responses [18,20,21,26,34,35,50,62,84,94]. Future research could expand on our work by comparing potential moderating effects of a wider range of coping styles, such as social support seeking or mindfulness, in order to identify and ultimately cultivate the most cardio-protective behaviors to buffer the effects of racial discrimination among this group. Finally, the goal of the present research was to better understand the unique life experiences of US-born African American women, which may be distinct from the experiences of Blacks who grew up in different social and racial contexts. Future work may consider whether these associations and interactions persist among African American men and in other Black groups such as Afro-Caribbeans and continental Africans.

## 5. Conclusions

The results of this study suggest that chronic exposure to racial discrimination, coupled with active coping responses to racism-specific stressors, may increase hypertension risk among African American women. By delineating coping dispositions from contextual coping behaviors, this study provides key insight into the stress-response process, which, if confirmed, can inform more targeted interventions to buffer the health consequences of racial discrimination among African American women. Specifically, interventions aimed at modifying the John Henryism coping disposition are likely less feasible than those modifying racism-specific active coping behavior, given John Henryism is a stable personality trait rather than a flexible response to environmental stimuli. Moreover, based on the results of this study, interventions on John Henryism may do little to mitigate the deleterious effects of racial discrimination among African American women. In contrast, the application of cognitive behavioral [95], mindfulness-based [96], or other modalities to cultivate and nurture adaptive racism-specific coping behaviors may be a feasible and effective strategy for attenuating the association between racial discrimination and hypertension among African American women. Results also suggest that efforts to intervene on coping behavior must be tailored to the chronicity of racial discrimination experienced. Among those experiencing more acute discrimination events, active coping behavior may be adaptive, whereas those navigating more chronic discrimination may benefit from a different set of coping approaches. Ultimately, however, sustainable change necessitates intervening on the root cause of African American women’s exposure to racial discrimination and associated stress by working to dismantle structural, institutional, and ideological racism as a *Fundamental Cause* of racial health inequities [97].

## Figures and Tables

**Table 1:** Everyday Discrimination Scale item weighting structure and categorical levels, African American Women’s Heart and Health Study, Northern California (n = 207).

Original Survey Item	Weight ^1^
Never	0
Less than once a year	0.5
A few times a year	3
A few times a month	36
At least once a week	104
Almost everyday	260
**Summary Score**	**Range ^1^**
	0–2600
**Categorical Level**	**Cutpoint ^1^**
Yearly or less: <12 x/year	<12
Monthly: ≥1 x/month and <1 x/week	≥12 and <52
Weekly: ≥1 x/week and <1 x/day	≥52 and <365
Daily: ≥1 x/day and <3 x/day	≥365 and <1095
Hourly: ≥3 x/day	≥1095

^1^ Values represent the total number of annual experiences across 10 distinct instances of everyday racial discrimination (see Supplemental Table S1 for index of EDS survey items).

**Table 2:** Characteristics of study sample, African American Women’s Heart and Health Study (n = 207).

Basic Demographics	n	%
Age (mean/SD)	41.73	5.90
Not married/partnered	146	70.53
**Socioeconomic factors**	**n**	**%**
In poverty: ≤100% FPL	39	18.84
≤High school diploma	69	33.33
Unemployed	93	44.93
**Biologic and behavioral factors**	**n**	**%**
Current smoker	89	43.00
≥3 drinks/day	38	18.36
Exercise <5 times/week	133	64.25
BMI < 18.5 or ≥25	179	86.47
Currently taking CV medication	43	20.77
**Blood pressure outcomes**	**mean**	**SD**
Systolic blood pressure	122.09	19.85
Diastolic blood pressure	80.37	11.66
Hypertensive ^1^ (n/%)	112	54.11
**Everyday Racial Discrimination (EDS)**	**n**	**%**
Yearly or less	48	23.19
Monthly	38	18.36
Weekly	50	24.15
Daily	36	17.39
Hourly	35	16.91
**Active coping**	**mean**	**SD**
John Henryism (mean/SD)	49.71	7.28
Active coping with racism (mean/SD)	8.98	2.32

^1^ Hypertensive if: (a) systolic blood pressure ≥ 130 mmHg or (b) diastolic blood pressure ≥ 80 mmHg or (c) self-reported current cardiovascular medication use. Abbreviations: SD = standard deviation, FPL = federal poverty level, BMI = body mass index, CV = cardiovascular, EDS = Everyday Discrimination Scale. Covariate reference categories: married/partnered, >100% FPL, >high school diploma, employed, nonsmoker or former smoker, <3 drinks/day, exercise ≥ 5 times/week, recommended BMI (≥18.5 and <25), and not currently taking CV medication.

**Table 3:** Prevalence ratios (PRs) and 95% confidence intervals (CIs) for main associations and interactions between everyday racial discrimination (EDS) and John Henryism active coping disposition (JH) on prevalence of hypertension, African American Women’s Heart and Health Study (n = 207).

	Model 1: Main Associations for EDS		Model 2: Main Associations for JH		Model 3: Interaction of EDS and JH	
	PR	95% CI	PR	95% CI	PR	95% CI
**Everyday Racial Discrimination (EDS) ^1^**						
Monthly	0.98	0.63, 1.53			0.97	0.62, 1.53
Weekly	1.31	0.90, 1.93			1.30	0.89, 1.90
Daily	1.26	0.86, 1.84			1.26	0.86, 1.85
Hourly	0.95	0.58, 1.56			0.93	0.56, 1.54
**John Henryism (JH) ^2^**			0.99	0.98, 1.01	0.99	0.96, 1.01
EDS*JH Interactions						
Monthly*JH					1.02	0.96, 1.09
Weekly*JH					1.01	0.97, 1.05
Daily*JH					1.00	0.97, 1.04
Hourly*JH					0.99	0.95, 1.03
**Model F-Test ^3^**	F(13, 6330) = 3.12, p = 0.00		F(10, 11161) = 3.41, p = 0.00		F(18, 7368) = 2.29, p = 0.00	
**Interaction F-Test**	N/A		N/A		F(4, 51397) = 0.27, p = 0.90	

^1^ Referent group = EDS experienced yearly or less. ^2^ Mean-centered. ^3^ Overall joint test of interaction (two-tailed). * Denotes multiplicative interaction term in regression model. Abbreviations: EDS = Everyday Discrimination Scale, JH = John Henryism, PR = prevalence ratio, CI = confidence interval. Models adjust for: age, marital/partnership status, income, education, employment, BMI, smoking, drinking, and exercise.

**Table 4:** Prevalence ratios (PRs) and 95% confidence intervals (CIs) for main associations and interactions between everyday racial discrimination (EDS) and active coping with racism (ACR) on prevalence of hypertension, African American Women’s Heart and Health Study (n = 207).

	Model 4: Main Associations for ACR		Model 5: Interaction of EDS and ACR	
	PR	95% CI	PR	95% CI
**Everyday Racial Discrimination (EDS) ^1^**				
Monthly			1.03	0.58, 1.80
Weekly			1.53	0.98, 2.39
Daily			1.49	0.94, 2.36
Hourly			1.14	0.66, 1.96
**Active Coping with Racism (ACR) ^2^**	0.96	0.91, 1.04	0.96	0.91, 1.01
EDS*ACR Interactions				
Monthly*ACR			1.32	1.08, 1.61
Weekly*ACR			1.12	0.97, 1.30
Daily*ACR			1.17	1.01, 1.35
Hourly*ACR			1.11	0.93, 1.33
**Model F-Test ^3^**	F(10, 10060) = 3.31, p = 0.00		F(18, 3653) = 2.71, p = 0.00	
**Interaction F-Test**	N/A		F(4, 6495) = 2.33, p = 0.05	

^1^ Referent group = EDS experienced yearly or less. ^2^ Mean-centered. ^3^ Overall joint test of interaction (two-tailed). * Denotes multiplicative interaction term in regression model. Abbreviations: EDS = Everyday Discrimination Scale, ACR = Active Coping with Racism, FPL = federal poverty level, BMI = body mass index, PR = prevalence ratio, CI = confidence interval. Models adjust for: age, marital/partnership status, income, education, employment, BMI, smoking, drinking, and exercise.

**Table 5:** Prevalence ratios (PRs) and 95% confidence intervals (CIs) for associations between everyday racial discrimination (EDS) and hypertension at minimum, median, and maximum values of active coping with racism (ACR), African American Women’s Heart and Health Study (n = 207).

	Low ACR ^1^		Moderate ACR ^2^		High ACR ^3^	
	PR	95% CI	PR	95% CI	PR	95% CI
**Everyday Racial Discrimination (EDS) ^4^**						
Monthly	0.20	0.05, 0.83	1.03	0.58, 1.80	2.35	1.13, 4.87
Weekly	0.77	0.35, 1.70	1.53	0.98, 2.39	2.15	1.01, 4.61
Daily	0.60	0.25, 1.42	1.49	0.94, 2.36	2.36	1.14, 4.88
Hourly	0.60	0.21, 1.74	1.14	0.66, 1.96	1.57	0.66, 3.74

^1^ Low ACR = 3 (minimum score). ^2^ Low ACR = 9 (median score). ^3^ High ACR = 12 (maximum score). ^4^ Referent group = EDS experienced yearly or less. Abbreviations: ACR = Active Coping with Racism, EDS = Everyday Discrimination Scale, PR = prevalence ratio, CI = confidence interval. Models adjust for: age, marital/partnership status, income, education, employment, BMI, smoking, drinking, and exercise.

## Supplementary Materials

The following are available online at https://www.mdpi.com/1660-4601/16/23/4759/s1, Table S1. Everyday Discrimination Scale (EDS) item response distribution (n (%)) and summary score (range, mean(SD) median(IQR)), African American Women’s Heart and Health Study (n = 207), Table S2. John Henryism Active Coping (JHAC12) scale item response distribution (n (%)) and summary score (range, mean(SD) median(IQR)), African American Women’s Heart and Health Study (n = 207), Table S3. Active Coping with Racism (ACR) item response distribution (n (%)) and summary score (range, mean(SD) median(IQR)), African American Women’s Heart and Health Study (n = 207), Table S4. Associations of study variables, African American Women’s Heart and Health Study (n = 207), Table S5. Prevalence ratios (PRs) and 95% confidence intervals (CIs) for main associations and interactions between everyday racial discrimination (EDS) and John Henryism active coping disposition (JH) on prevalence of hypertension (including estimates for model covariates), African American Women’s Heart and Health Study (n = 207), Table S6. Prevalence ratios (PRs) and 95% confidence intervals (CIs) for main associations and interactions between everyday racial discrimination (EDS) and active coping with racism (ACR) on prevalence of hypertension (including estimates for model covariates), African American Women’s Heart and Health Study (n = 207).
