# Supplementary material for: Everyday Racial Discrimination and Hypertension among Midlife African American Women: Disentangling the Role of Active Coping Dispositions versus Active Coping Behaviors
Source: Int J Environ Res Public Health. 2019 Nov 27;16(23):4759. doi: 10.3390/ijerph16234759 (PMC6935759; doi:10.3390/ijerph16234759)
Supplement: Supplementary file 1 [file ijerph-16-04759-s001.zip › ijerph-612355- supplementary tables_final/Supplementary_TableS6.docx]

**Supplemental Table S6.** Prevalence ratios (PRs) and 95% confidence intervals (CIs) for main associations and interactions between everyday racial discrimination (EDS) and active coping with racism (ACR) on prevalence of hypertension (including estimates for model covariates), African American Women’s Heart & Health Study (n=207)

|  | **Model 4:**  **Main Associations for ACR** | | **Model 5:**  **Interaction of EDS and ACR** | |
| --- | --- | --- | --- | --- |
|  | PR | 95% CI | PR | 95% CI |
| **Everyday Racial Discrimination (EDS) ^1^** |  |  |  |  |
| Monthly |  |  | 1.03 | 0.58, 1.80 |
| Weekly |  |  | 1.53 | 0.98, 2.39 |
| Daily |  |  | 1.49 | 0.94, 2.36 |
| Hourly |  |  | 1.14 | 0.66, 1.96 |
| **Active Coping with Racism (ACR) ^2^** | 0.96 | 0.91, 1.04 | 0.96 | 0.91, 1.01 |
| **EDS*ACR Interactions** |  |  |  |  |
| Monthly*ACR |  |  | 1.32 | 1.08, 1.61 |
| Weekly*ACR |  |  | 1.12 | 0.97, 1.30 |
| Daily*ACR |  |  | 1.17 | 1.01, 1.35 |
| Hourly*ACR |  |  | 1.11 | 0.93, 1.33 |
| **Covariates** |  |  |  |  |
| Age | 1.05 | 1.03, 1.08 | 1.05 | 1.02, 1.08 |
| Not married/partnered | 0.79 | 0.61, 1.02 | 0.79 | 0.60, 1.04 |
| In poverty: ≤ 100% FPL | 1.13 | 0.79, 1.60 | 1.14 | 0.81, 1.62 |
| ≤ High school diploma | 0.97 | 0.73, 1.27 | 1.02 | 0.77, 1.34 |
| Unemployed | 1.04 | 0.79, 1.34 | 1.01 | 0.77, 1.33 |
| Current smoker | 1.29 | 0.96, 1.71 | 1.23 | 0.92, 1.66 |
| ≥ 3 drinks/day | 1.09 | 0.79, 1.48 | 1.11 | 0.80, 1.53 |
| Exercise < 5 times/week | 1.29 | 0.96, 1.71 | 1.27 | 0.94, 1.71 |

| BMI < 18.5 or ≥ 25 | 1.30 | 0.89, 1.89 | 1.30 | 0.09, 1.90 |
| --- | --- | --- | --- | --- |
| **Model F-Test ^3^** | F(10, 10060) = 3.31, p=0.00 | | F(18, 3653) = 2.71, p = 0.00 | |
| **Interaction F-Test** | N/A | | F(4, 6495) = 2.33, p = 0.05 | |

^1^ Referent group = EDS experienced yearly or less.

^2^ Mean-centered.

^3^ Overall joint test of interaction (two-tailed).

* Denotes multiplicative interaction term in regression model.

Abbreviations: EDS = Everyday Discrimination Scale, ACR = Active Coping with Racism, FPL = federal poverty level, BMI = body mass index, PR = prevalence ratio, CI = confidence interval.

Reference categories: yearly EDS, married/partnered, > 100% FPL, > high school diploma, employed, nonsmoker or former smoker, < 3 drinks/day, exercise ≥ 5 times/week, recommended BMI (≥ 18.5 and < 25).
