# Supplementary material for: Everyday Racial Discrimination and Hypertension among Midlife African American Women: Disentangling the Role of Active Coping Dispositions versus Active Coping Behaviors
Source: Int J Environ Res Public Health. 2019 Nov 27;16(23):4759. doi: 10.3390/ijerph16234759 (PMC6935759; doi:10.3390/ijerph16234759)
Supplement: Supplementary file 1 [file ijerph-16-04759-s001.zip › ijerph-612355- supplementary tables_final/Supplementary_TableS1.docx]

**Supplemental Table S1.** Everyday Discrimination Scale (EDS) item response distribution (n(%)) and summary score (range, mean(SD) median(IQR)), African American Women’s Heart & Health Study (n=207)

| **EDS survey item ^1^** | **Never** | **Less than once a year** | **A few times a year** | **A few times a month** | **At least once a week** | **Almost everyday** |
| --- | --- | --- | --- | --- | --- | --- |
| You are treated with less courtesy than other people | 24 (11.59) | 27 (13.04) | 64 (30.92) | 39 (18.84) | 27 (13.04) | 26 (12.56) |
| You are treated with less respect than other people | 34 (16.43) | 30 (14.49) | 57 (27.54) | 33 (15.94) | 25 (12.08) | 28 (13.53) |
| You receive poorer service than other people at restaurants or stores | 26 (12.56) | 42 (20.29) | 60 (28.99) | 37 (17.87) | 19 (9.18) | 23 (11.11) |
| People act as if they think you are not smart | 50 (24.15) | 34 (16.43) | 48 (23.19) | 22 (10.63) | 27 (13.04) | 26 (12.56) |
| People act as if they are afraid of you | 68 (32.85) | 34 (16.43) | 31 (14.98) | 26 (12.56) | 23 (11.11) | 25 (12.08) |
| People act as if you are dishonest | 65 (31.40) | 41 (19.81) | 38 (18.36) | 21 (10.14) | 14 (6.76) | 28 (13.53) |
| People act as if they’re better than you are | 26 (12.56) | 25 (12.08) | 45 (21.74) | 41 (19.81) | 23 (11.11) | 47 (13.00) |
| You are called names or insulted | 95 (45.89) | 40 (19.32) | 34 (16.43) | 12 (5.80) | 13 (6.28) | 13 (6.28) |
| You are threatened or harassed | 118 (57.00) | 32 (15.46) | 24 (11.59) | 10 (4.83) | 9 (4.35) | 14 (6.76) |
| You are followed around in stores | 45 (21.74) | 38 (18.36) | 62 (29.95) | 18 (8.70) | 18 (8.70) | 26 (12.56) |
| **EDS summary score** | **Range** | **Mean (SD)** | **Median (IQR)** |  |  |  |
|  | 0, 2600 | 473.61 (695.05) | 186.50 (16, 666) |  |  |  |

^1^ Survey question asks: “In your day-to-day life, how often have any of the following things happened to you because of your race, ethnicity, or skin color?”

Abbreviations: EDS = Everyday Discrimination Scale; SD = standard deviation.
