# Supplementary material for: Everyday Racial Discrimination and Hypertension among Midlife African American Women: Disentangling the Role of Active Coping Dispositions versus Active Coping Behaviors
Source: Int J Environ Res Public Health. 2019 Nov 27;16(23):4759. doi: 10.3390/ijerph16234759 (PMC6935759; doi:10.3390/ijerph16234759)
Supplement: Supplementary file 1 [file ijerph-16-04759-s001.zip › ijerph-612355- supplementary tables_final/Supplementary_TableS2.docx]

**Supplemental Table S2.** John Henryism Active Coping (JHAC12) scale item response distribution (n(%)) and summary score (range, mean(SD) median(IQR)), African American Women’s Heart & Health Study (n=207)

| **JHAC12 survey item ^1^** | **Completely false** | **Somewhat false** | **Don't know** | **Somewhat true** | **Completely true** |
| --- | --- | --- | --- | --- | --- |
| I've always felt that I could make my life pretty much what I wanted to make of it. | 4 (1.93) | 12 (5.80) | 17 (8.21) | 71 (34.30) | 103 (49.76) |
| Once I make up my mind to do something, I stay with it until the job is completely done. | 2 (0.97) | 22 (10.63) | 10 (4.83) | 75 (36.23) | 98 (47.34) |
| I like doing things that other people thought could not be done. | 6 (2.90) | 11 (5.31) | 18 (8.70) | 73 (35.27) | 99 (47.83) |
| When things don't go the way I want them to, that just makes me work even harder. | 4 (1.93) | 16 (7.73) | 16 (7.73) | 85 (41.06) | 86 (41.55) |
| Sometimes I feel that if anything is going to be done right, I have to do it myself. | 3 (1.45) | 8 (3.86) | 13 (6.28) | 66 (31.88) | 117 (56.52) |
| It's not always easy, but I manage to find a way to do the things that I really need to get done. | 2 (0.97) | 4 (1.93) | 11 (5.31) | 55 (26.57) | 135 (65.22) |
| Very seldom have I been disappointed by the results of my hard work. | 13 (6.28) | 30 (14.49) | 13 (6.28) | 100 (48.31) | 31 (24.64) |
| I feel that I am the kind of individual who stands up for what she believes in, regardless of the consequences. | 2 (0.97) | 6 (2.90) | 12 (5.80) | 88 (42.51) | 99 (47.83) |
| In the past, even when things got really tough, I never lost sight of my goals. | 6 (2.90) | 25 (12.08) | 13 (6.28) | 88 (42.51) | 75 (36.23) |
| It's important for me to be able to do things the way I want to do them rather than the way other people want me to do them. | 3 (1.45) | 24 (11.59) | 13 (6.28) | 91 (43.96) | 76 (36.71) |
| I don't let my personal feelings get in the way of doing a job. | 11 (5.31) | 31 (14.98) | 13 (6.28) | 83 (40.10) | 69 (33.33) |
| Hard work has really helped me to get ahead in life. | 6 (2.90) | 10 (4.83) | 20 (9.66) | 73 (35.27) | 98 (47.34) |
| **JHAC12 summary score** | **Range** | **Mean (SD)** | **Median (IQR)** |  |  |
|  | 16, 60 | 49.71 (7.28) | 51 (46, 55) |  |  |

^1^ Survey question asks: “The following are some statements about how you see yourself right now, today, living and doing things in the real world. Please indicate how true or false these statements are for you, personally.”

Abbreviations: JHAC12 = John Henryism Active Coping 12 Scale, SD=standard deviation
