# Supplementary material for: Everyday Racial Discrimination and Hypertension among Midlife African American Women: Disentangling the Role of Active Coping Dispositions versus Active Coping Behaviors
Source: Int J Environ Res Public Health. 2019 Nov 27;16(23):4759. doi: 10.3390/ijerph16234759 (PMC6935759; doi:10.3390/ijerph16234759)
Supplement: Supplementary file 1 [file ijerph-16-04759-s001.zip › ijerph-612355- supplementary tables_final/Supplementary_TableS3.docx]

**Supplemental Table S3.** Active Coping with Racism (ACR) item response distribution (n (%)) and summary score (range, mean(SD) median(IQR)), African American Women’s Heart & Health Study (n=207)

| **ACR survey item ^1^** | **Never** | **Rarely** | **Some of the time** | **Most of the time** |
| --- | --- | --- | --- | --- |
| Speak up or try do something about it | 14 (6.76) | 34 (11.59) | 75 (36.23) | 94 (45.41) |
| Work harder to try to change the situation | 24 (11.59) | 31 (14.98) | 93 (44.93) | 59 (25.80) |
| Work harder to try to prove them wrong | 29 (14.01) | 37 (17.87) | 72 (34.78) | 69 (66.67) |
| **ACR summary score** | **Range** | **Mean (SD)** | **Median (IQR)** |  |
|  | 3, 12 | 8.98 (2.32) | 9 (8, 11) |  |

^1^ Survey question asks: “The following are some statements about how you see yourself right now, today, living and doing things in the real world. Please indicate how true or false these statements are for you, personally.”

Abbreviations: ACR = Active Coping with Racism; SD = standard deviation.
