# Supplementary material for: Everyday Racial Discrimination and Hypertension among Midlife African American Women: Disentangling the Role of Active Coping Dispositions versus Active Coping Behaviors
Source: Int J Environ Res Public Health. 2019 Nov 27;16(23):4759. doi: 10.3390/ijerph16234759 (PMC6935759; doi:10.3390/ijerph16234759)
Supplement: Supplementary file 1 [file ijerph-16-04759-s001.zip › ijerph-612355- supplementary tables_final/Supplementary_TableS4.docx]

**Supplemental Table S4.** Associations of Study Variables, African American Women’s Heart & Health Study (n=207)

| **Study Variable** | **1** | **2** | **3** | **4** | **5** | **6** | **7** | **8** | **9** | **10** | **11** | **12** | **13** | **14** | **15** | **16** |
| --- | --- | --- | --- | --- | --- | --- | --- | --- | --- | --- | --- | --- | --- | --- | --- | --- |
| 1. **Age** | --- | t_(205)_=0.77,  p=0.44 | t_(205)_=0.70  p=0.49 | t_(205)_=-0.77  p=0.44 | t_(205)_=-0.70  p=0.48 | t_(205)_=0.65  p=0.51 | t_(205)_=1.48  p=0.14 | t_(205)_=0.06  p=0.95 | t_(205)_=-0.46  p=0.65 | t_(205)_=-3.42  p=0.00 | r=0.30 | r=0.23 | t_(205)_=-4.07  p=0.000 | r=0.02 | r=0.03 | F_(206)_=20.3  p=0.09 |
| 1. **Not married/partnered** |  | --- | χ^2^_(1)_=0.34  p=0.56 | χ^2^_(1)_=1.41  p=0.24 | χ^2^_(1)_=4.09  p=0.04 | χ^2^_(1)_=11.01  p=0.00 | χ^2^_(1)_=0.006  p=0.94 | χ^2^_(1)_=1.78  p=0.18 | χ^2^_(1)_=0.25  p=0.62 | χ^2^_(1)_=4.01  p=0.05 | t_(205)_=1.59  p=0.11 | t_(205)_=2.02  p=0.04 | χ^2^_(1)_=3.36  p=0.07 | t_(205)_=0.71  p=0.48 | t_(205)_=1.26  p=0.10 | χ^2^_(4)_=3.97  p=0.41 |
| 1. **In poverty: ≤ 100%FPL** |  |  | --- | χ^2^_(1)_=0.57  p=0.45 | χ^2^_(1)_=1.55  p=0.21 | χ^2^_(1)_=5.00  p=0.03 | χ^2^_(1)_=0.15  p=0.70 | χ^2^_(1)_=13.92  p=0.00 | χ^2^_(1)_=1.79  p=0.18 | χ^2^_(1)_=0.00  p=0.97 | t_(205)_=-0.02  p=0.98 | t_(205)_=-0.39  p=0.70 | χ^2^_(1)_=0.46  p=0.50 | t_(205)_=1.80  p=0.07 | t_(205)_=0.33  p=0.75 | χ^2^_(4)_=0.07  p=1.0 |
| 1. **≤ High school diploma** |  |  |  | --- | χ^2^_(1)_=14.85  p=0.00 | χ^2^_(1)_=18.22  p=0.00 | χ^2^_(1)_=1.61  p=0.20 | χ^2^_(1)_=0.04  p=0.84 | χ^2^=0.18  p=0.68 | χ^2^_(1)_=0.02  p=0.90 | t_(205)_=-1.32  p=0.19 | t_(205)_=-2.14  p=0.03 | χ^2^_(1)_=1.18  p=0.28 | t_(205)_=0.70  p=0.48 | t_(205)_=2.89  p=0.00 | χ^2^_(4)_=11.85  p=0.02 |
| 1. **Unemployed** |  |  |  |  | --- | χ^2^_(1)_=25.85  p=0.00 | χ^2^_(1)_=0.48  p=0.49 | χ^2^_(1)_=3.88  p=0.05 | χ^2^_(1)_=0.04  p=0.85 | χ^2^_(1)_=0.06  p=0.82 | t_(205)_=-0.73  p=0.47 | t_(205)_=-0.79  p=0.43 | χ^2^_(1)_=1.07  p=0.30 | t_(205)_=1.67  p=0.10 | t_(205)_=-0.23  p=0.82 | χ^2^_(4)_=1.77  p=0.78 |
| 1. **Current smoker** |  |  |  |  |  | --- | χ^2^_(1)_=1.76  p=0.18 | χ^2^_(1)_=8.90  p=0.00 | χ^2^_(1)_=0.19  p=0.66 | χ^2^_(1)_=0.27  p=0.60 | t_(205)_=-1.76  p=0.08 | t_(205)_=-2.34  p=0.02 | χ^2^_(1)_=3.72  p=0.05 | t_(205)_=1.86  p=0.07 | t_(205)_=0.44  p=0.66 | χ^2^_(4)_= 6.32  p=0.18 |
| 1. **≥ 3 drinks/day** |  |  |  |  |  |  | --- | χ^2^_(1)_=0.82  p=0.37 | χ^2^_(1)_=1.64  p=0.20 | χ^2^_(1)_=5.11  p=0.024 | t_(205)_=-0.84  p=0.80 | t_(205)_=-0.85  p=0.40 | χ^2^_(1)_=0.03  p=0.87 | t=-0.33  p=0.75 | t_(205)_=0.87  p=0.38 | χ^2^_(4)_=8.56  p=0.07 |
| 1. **Exercise < 5 times/week** |  |  |  |  |  |  |  | --- | χ^2^_(1)_=0.28  p=0.60 | χ^2^_(1)_=0.02  p=0.89 | t_(205)_=-0.74  p=0.460 | t_(205)_=-0.54  p=0.59 | χ^2^_(1)_=1.38  p=0.24 | t_(205)_=-0.28  p=0.78 | t_(205)_=-1.04  p=0.30 | χ^2^_(4)_=2.87  p=0.58 |
| 1. **BMI < 18.5 or ≥ 25** |  |  |  |  |  |  |  |  | --- | χ^2^_(1)_=0.14  p=0.71 | t_(205)_=-1.27  p=0.21 | t_(205)_=-2.28  p=0.02 | χ^2^_(1)_=2.81  p=0.09 | t_(205)_=0.48  p=0.63 | t_(205)_=-0.38  p=0.71 | χ^2^_(4)_=3.90  p=0.42 |
| 1. **Currently taking**   **CV medication** |  |  |  |  |  |  |  |  |  | --- | t_(205)_=-3.42  p=0.00 | t_(205)_=-4.00  p=0.00 | χ^2^_(1)_=46.04  p=0.00 | t_(205)_=0.31  p=0.76 | t_(205)_=-0.06  p=0.95 | χ^2^_(4)_=2.14  p=0.71 |
| 1. **Systolic blood pressure** |  |  |  |  |  |  |  |  |  |  | --- | r=0.79 | t_(205)_=-12.19  p=0.00 | r=0.05 | r=0.01 | F_(206)_=0.87  p=0.48 |
| 1. **Diastolic blood pressure** |  |  |  |  |  |  |  |  |  |  |  |  | t_(205)_=-14.75  p=0.00 | r=-0.04 | r=-0.04 | F_(206)_=0.44  p=0.78 |
| 1. **Hypertensive ^1^** |  |  |  |  |  |  |  |  |  |  |  |  | --- | t_(205)_=1.23  p=0.22 | t_(205)_=0.77  p=0.44 | χ^2^_(4)_=8.53  p=0.07 |
| 1. **John Henryism** |  |  |  |  |  |  |  |  |  |  |  |  |  | --- | r=0.26 | F_(206)_=0.60  p=0.66 |
| 1. **Active Coping with Racism** |  |  |  |  |  |  |  |  |  |  |  |  |  |  | --- | F_(206)_=1.66  p=0.16 |
| 1. **Everyday Discrimination**   **Scale ^2^** |  |  |  |  |  |  |  |  |  |  |  |  |  |  |  | --- |

^1^ Hypertensive if: (a) systolic blood pressure ≥ 130 mmHg or (b) diastolic blood pressure ≥ 80 mmHg or (c) self-reported current cardiovascular medication use

^2^ Categorical measure of EDS (yearly or less, monthly, weekly, daily, hourly).

Abbreviations: FPL = federal poverty level, BMI = body mass index, CV = cardiovascular.

Reference categories: married/partnered, > high school diploma, employed, nonsmoker or former smoker, < 3 drinks/day, exercise ≥ 5 times/week, recommended BMI (≥ 18.5 and < 25), not currently taking CV medication, not hypertensive.

Note: ttests are two-tailed, values in parentheses are degrees of freedom.
