# Supplementary material for: Everyday Racial Discrimination and Hypertension among Midlife African American Women: Disentangling the Role of Active Coping Dispositions versus Active Coping Behaviors
Source: Int J Environ Res Public Health. 2019 Nov 27;16(23):4759. doi: 10.3390/ijerph16234759 (PMC6935759; doi:10.3390/ijerph16234759)
Supplement: Supplementary file 1 [file ijerph-16-04759-s001.zip › ijerph-612355- supplementary tables_final/Supplementary_TableS5.docx]

**Supplemental Table S5.** Prevalence ratios (PRs) and 95% confidence intervals (CIs) for main associations and interactions between everyday racial discrimination (EDS) and John Henryism active coping disposition (JH) on prevalence of hypertension (including estimates for model covariates), African American Women’s Heart & Health Study (n=207)

|  | **Model 1:**  **Main Associations for EDS** | | **Model 2:**  **Main Associations for JH** | | **Model 3:**  **Interaction of EDS and JH** | |
| --- | --- | --- | --- | --- | --- | --- |
|  | **PR** | **95% CI** | **PR** | **95% CI** | **PR** | **95% CI** |
|  | | | | | | |
| **Everyday Racial Discrimination (EDS) ^1^** |  |  |  |  |  |  |
| Monthly | 0.98 | 0.63, 1.53 |  |  | 0.97 | 0.62, 1.53 |
| Weekly | 1.31 | 0.90, 1.93 |  |  | 1.30 | 0.89, 1.90 |
| Daily | 1.26 | 0.86, 1.84 |  |  | 1.26 | 0.86, 1.85 |
| Hourly | 0.95 | 0.58, 1.56 |  |  | 0.93 | 0.56, 1.54 |
| **John Henryism (JH) ^2^** |  |  | 0.99 | 0.98, 1.01 | 0.99 | 0.96, 1.01 |
| **EDS*JH Interactions** |  |  |  |  |  |  |
| Monthly*JH |  |  |  |  | 1.02 | 0.96, 1.09 |
| Weekly*JH |  |  |  |  | 1.01 | 0.97, 1.05 |
| Daily*JH |  |  |  |  | 1.00 | 0.97, 1.04 |
| Hourly*JH |  |  |  |  | 0.99 | 0.95, 1.03 |
| **Covariates** |  |  |  |  |  |  |
| Age | 1.05 | 1.03, 1.08 | 1.05 | 1.03, 1.08 | 1.05 | 1.02, 1.08 |
| Not married/partnered | 0.81 | 0.63, 1.05 | 0.79 | 0.62, 1.03 | 0.79 | 0.61, 1.03 |
| In poverty: ≤ 100%FPL | 1.14 | 0.80, 1.63 | 1.12 | 0.79, 1.60 | 1.12 | 0.78, 1.60 |
| ≤ High school diploma | 1.02 | 0.77, 1.34 | 1.01 | 0.77, 1.31 | 1.00 | 0.76, 1.32 |
| Unemployed | 0.99 | 0.75, 1.29 | 1.00 | 0.77, 1.30 | 0.99 | 0.75, 1.31 |
| Current smoker | 1.26 | 0.94, 1.68 | 1.25 | 0.94, 1.67 | 1.24 | 0.92, 1.66 |
| ≥ 3 drinks/day | 1.09 | 0.79, 1.50 | 1.10 | 0.80, 1.52 | 1.11 | 0.79, 1.55 |
| Exercise < 5 times/week | 1.25 | 0.93, 1.66 | 1.26 | 0.95, 1.67 | 1.24 | 0.93, 1.64 |
| BMI < 18.5 or ≥ 25 | 1.28 | 0.88, 1.86 | 1.27 | 0.87, 1.84 | 1.27 | 0.88, 1.85 |
| **Model F-Test ^3^** | F(13, 6330)=3.12, p=0.00 | | F(10, 11161)=3.41, p=0.00 | | F(18, 7368)=2.29, p=0.00 | |
| **Interaction F-Test** | N/A | |  | | F(4, 51397)=0.27, p=0.90 | |

^1^ Referent group = EDS experienced yearly or less.

^2^ Mean-centered.

^3^ Overall joint test of interaction (two-tailed).

* Denotes multiplicative interaction term in regression model.

Abbreviations: EDS = Everyday Discrimination Scale, JH = John Henryism, BMI = body mass index, HS = high school, PR = prevalence ratio, CI = confidence interval.

Reference categories: yearly EDS, married/partnered, > 100% FPL, > high school diploma, employed, nonsmoker or former smoker, < 3 drinks/day, exercise ≥ 5 times/week, recommended BMI (≥ 18.5 and < 25).
